# The Implementation of Trifecta Score to Assess the Quality of Holmium Laser Enucleation of the Prostate in Elderly Patients: An Analysis of Perioperative and Functional Outcomes and the Impact of Age

**DOI:** 10.3390/jcm14103410

**Published:** 2025-05-13

**Authors:** Carlo Giulioni, Matteo Tallè, Alessio Papaveri, Francesco Mengoni, Roberto Orciani, Savio Domenico Pandolfo, Ciro Imbimbo, Felice Crocetto, Valentina Maurizi, Vineet Gauhar, Angelo Cafarelli

**Affiliations:** 1 Department of Urology, Casa di Cura Villa Igea, 60127 Ancona, Italy; mtalle@casadicuravillaigea.it (M.T.); apapaveri@casadicuravillaigea.it (A.P.); fmengoni@casadicuravillaigea.it (F.M.); rorciani@casadicuravillaigea.it (R.O.); info@angelocafarelli.it (A.C.); 2 Department of Urology, University of L’Aquila, 67100 L’Aquila, Italy; pandolfosavio@gmail.com; 3 Department of Neurosciences and Reproductive Sciences and Odontostomatology, University of Naples “Federico II”, 80131 Naples, Italy; ciro.imbimbo@unina.it (C.I.); felice.crocetto@unina.it (F.C.); 4 Internal Medicine Unit Jesi, Area Vasta 2 ASUR Marche, 60035 Jesi, Italy; valentina.b.maurizi@gmail.com; 5 Department of Urology, Ng Teng Fong General Hospital, Singapore 126817, Singapore; vineetgaauhaar@gmail.com

**Keywords:** trifecta, HoLEP, elderly, laser, benign prostatic hyperplasia

## Abstract

**Background**: The aim of this study was to assess the efficacy and safety of Holmium Laser Enucleation of the Prostate (HoLEP) in the treatment of symptomatic benign prostatic hyperplasia (BPH) in elderly patients using the Trifecta Score, based on a 1-year follow-up. **Methods**: We conducted a retrospective analysis of patients with BPH who underwent HoLEP at our institution between January 2016 and December 2022. The patients were divided into two groups: Group 1: patients aged ≥75 years, Group 2: patients aged below 74 years. The Trifecta Score achievement rates were then evaluated. Logistic regression analyses were performed to examine the impact of age on Trifecta parameters and to assess factors associated with urinary incontinence. **Results**: Overall, 981 participants were enrolled, with 490 in Group 1 and 491 in Group 2. Operative characteristics were similar between groups, though Group 1 had a longer time to catheter removal. At the 3-month follow-up, Group 1 had a higher IPSS and lower Qmax compared to Group 2, while there were no significant differences in functional outcomes by one year. In terms of postoperative morbidities, Group 1 exhibited a higher rate of blood transfusion. The Trifecta Score was similar between Groups 1 and 2 (63.5% vs. 68.8%, respectively, *p* = 0.08), and no parameter of that score exhibited a negative correlation with age. **Conclusions**: HoLEP demonstrates comparable functional outcomes to those observed in younger cohorts after one year. Overall, the Trifecta Score appears to be a valuable tool for this assessment. Nevertheless, incorporating an assessment of postoperative urinary continence and 1-year postoperative Qmax could enhance the system’s validity.

## 1. Introduction

Benign prostatic hyperplasia (BPH) is characterized by urethral compression and increased resistance during urination, leading to bladder outlet obstruction [1]. This results in a wide range of lower urinary tract symptoms (LUTS), which typically worsen gradually in severity. Notably, individuals with a prostate size exceeding 50 mL face a five-fold higher risk of experiencing clinically significant LUTS and a three-fold increased likelihood of substantial bladder outlet blockage [2]. Although the incidence of benign prostatic hyperplasia (BPH) is relatively low at 8% among individuals aged 40, it increases substantially to over 50% by age 60 and reaches approximately 83% in older adults [3]. Furthermore, BPH is characterized by a progressive increase in prostate volume with age, with an average annual growth rate estimated at around 1.6% [4].

Therefore, with prolonged life expectancy, the need for obstructive treatment for older patients who fail medical therapy is increasingly higher. According to the European Association of Urology (EAU) guidelines, Transurethral resection of the prostate (TURP) is considered the treatment of choice for moderate-to-severe LUTS in men with prostate sizes up to 80 mL, while open prostatectomy (OP) remains a valid option for larger prostates [5]. However, limitations regarding gland size and associated implications, such as long operative time (OT) and postoperative blood loss, may discourage its use in the elderly [6,7]. Holmium Laser Enucleation of the Prostate (HoLEP) has emerged as a safe surgical intervention for BPH, with its limited tissue penetration and effective hemostasis. In a recent review, no significant difference in perioperative complication rates was observed between age groups despite higher ASA scores and rates of anticoagulation/antiplatelet therapy in elderly patients [8]. The Trifecta Score is a comprehensive metric widely utilized in medical and surgical evaluations, particularly in urology, to assess the quality of surgical outcomes. However, its application in the field of endourology remains limited and not validated. In the context of BPH surgery, Autorino et al. proposed a Trifecta Score that considers the postoperative maximum urinary flow rate (Qmax), the International Prostate Symptom Score (IPSS), and the absence of perioperative complications [9]. Therefore, our aim is to evaluate how this Trifecta Score fares in elderly patients undergoing High-Power HoLEP for the treatment of symptomatic BPH in elderly patients at 1-year follow-up.

## 2. Materials and Methods

### 2.1. Patient Selection

A retrospective analysis of medical records was performed for all patients who underwent HoLEP at a single institution between January 2016 and December 2022. Preoperatively, all patients underwent transrectal ultrasound of the prostate, uroflowmetry, and serum prostate-specific antigen (PSA) measurement. The inclusion criteria were prostate volume exceeding 30 cc, PSA density of less than 0.20 ng/mL, Qmax below 15 mL/s, and IPSS greater than 7. Patients with incomplete data, PSA density exceeding 0.20 ng/mL, Qmax > 15 mL/s, and mild LUTS defined by an IPSS score of less than 7 were excluded.

The following demographic, functional and pathological characteristics were collected: age, Charlson Comorbidity Index (CCI) score, total serum PSA level, prostate volume, preoperatory indwelling urethral catheter, presence of bladder calculi, and history of medical or surgical management for BPH. Intra- and perioperative data, such as OT, enucleation time (ET), length of hospital stay (LOS), time to urethral catheter removal, postoperative complication within 30 days, were also collected. Early complications (within 30 days) were graded using the Clavien–Dindo (CD) classification. Functional outcomes, including IPSS and urodynamic parameters, were assessed at the 3-month and 1-year follow-up visits. As previously written, the Trifecta Score was assessed according to the criteria proposed by Autorino et al. [9], which includes the achievement of a postoperative Qmax exceeding 15 mL/s, an IPSS below 8, and the absence of perioperative complications.

Afterwards, stratification by age was employed to categorize the participants into two groups. Group 1 comprised individuals aged 75 years and above, while Group 2 included those younger than 75 years old. Although the inclusion of a frailty index would have been preferable, we selected an age threshold of 75 years as a pragmatic criterion for defining the elderly, as individuals in this age group typically exhibit multiple comorbidities and a greater need for assistance [10].

Formal ethics committee approval was deemed unnecessary for this type of study in our center because retrospective data collection was carried out for clinical purposes, and all the procedures were performed as part of routine care. All patients provided written informed consent for the collection of their anonymized data.

All procedures adhered to the ethical standards of the institutional and national research committees, as well as the 1964 Helsinki Declaration and its subsequent amendments or equivalent ethical guidelines.

### 2.2. Surgical Technique

All HoLEP procedures were performed following the technique described by Dr. P. J. Gilling et al. [11], which utilizes specific laser manipulation strategies to achieve efficient enucleation of the prostate tissue. The equipment utilized included a 120 W Holmium laser (Lumenis Inc.^®^, Palo Alto, CA, USA) with a reusable 550 nm laser fiber, a 26-Fr continuous flow resectoscope (Storz^®^), a 27-Fr nephroscope with a 5 mm working channel, a morcellator (Versacut, Lumenis^®^ Inc.), normal saline irrigation, and a video system.

After a small mucosal incision at the bladder neck, controlled dissection was performed using the laser fiber to create a working space between the prostatic capsule and the adenoma at the level of the prostatic apex. A “lateral-to-medial, apical-to-base” enucleation strategy was applied. Meticulous laser coagulation was performed throughout the procedure to ensure hemostasis. The enucleated adenoma was morcellated using the laser fiber into manageable fragments. In cases where adenomas with lobulated prostate tissue (“beach-balling”) were not effectively morcellated, monopolar resection was utilized for the fragmentation of the remaining tissue.

### 2.3. Statistical Analysis

Statistical analyses were performed using the IBM SPSS software package version 26.0 (IBM Corp., Armonk, NY, USA). Quantitative variables were described using means and standard deviations. Categorical variables were presented as frequencies and percentages. Continuous variables were compared between groups using the Mann–Whitney test, while the Chi-square test was employed to assess associations between categorical variables. A multivariate logistic regression analysis was performed to evaluate the influence of age in Trifecta Score and its parameters and to assess factors associated with UI. Data are presented as odds ratio (OR) and 95% confidence interval (CI). A *p*-value of less than 0.05 was considered statistically significant.

## 3. Results

**Figure 1:**
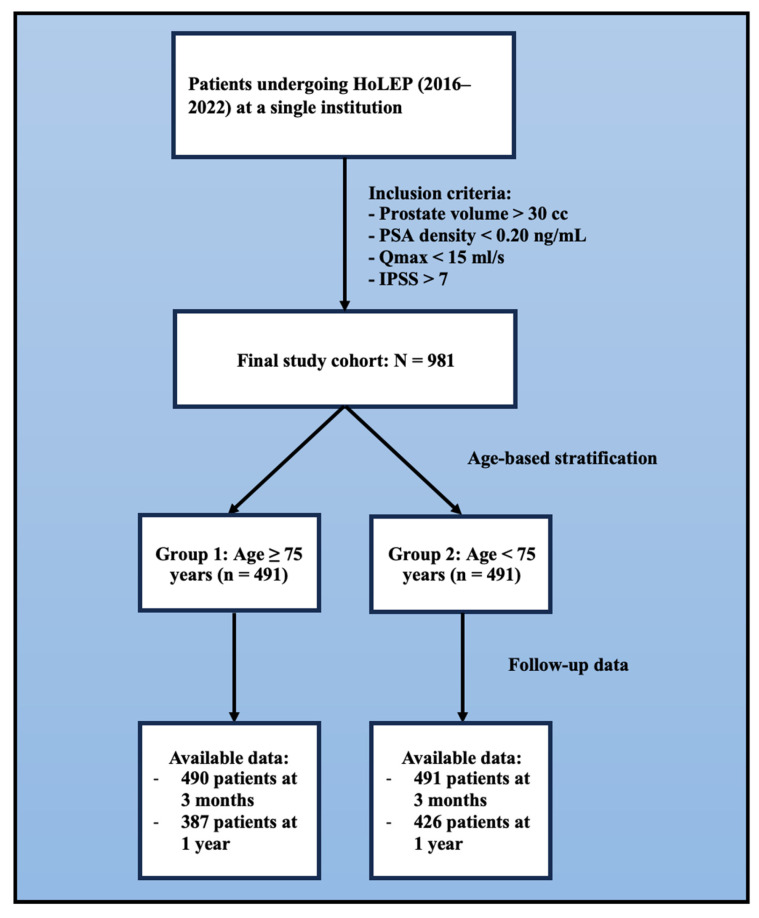
Flowchart of patient enrollment, stratification, and follow-up.

During the study period, 490 individuals were enrolled in Group 1 and 491 in Group 2, as shown in Figure 1.

Baseline demographics and characteristics related to BPH are detailed in Table 1. Group 1 exhibited significantly higher values in several measures compared to Group 2: BMI (26.8 vs. 25.3, *p* = 0.008), ASA score (1.87 vs. 1.79, *p* = 0.04), and CCI (4.43 vs. 1.75, *p* < 0.001). Additionally, a higher proportion of surgeries in Group 1 involved patients on antiplatelet or anticoagulant therapy (23.8% vs. 16.4%, *p* = 0.004). In terms of functional outcomes, Group 1 had a higher preoperative PVR volume compared to Group 2 (74.7 mL vs. 67.8 mL, *p* = 0.01), while other parameters were similar between the two groups.

Table 2 presents the operative characteristics. Statistical analysis indicated no significant differences between the two groups in terms of OT, ET, enucleation type, use of electrocautery after enucleation, and LOS. However, Group 1 experienced a longer time to urethral catheter removal (2.23 days vs. 2.07 days, *p* = 0.02) and a higher rate of incidental prostate cancer detection (5.5% vs. 2.7%, *p* = 0.04) compared to Group 2.

At the 3-month follow-up visit of 490 patients in Group 1 and 491 patients in Group 2, both groups showed a decrease in LUTS grade and improved uroflowmetry parameters. However, comparing the outcomes between Group 1 and Group 2, the PVR was similar (16.2 mL vs. 14.9 mL, *p* = 0.05), while Group 1 had a higher IPSS (7.6 vs. 7.0, *p* = 0.04) and a lower Qmax (23.6 mL/s vs. 27.9 mL/s, *p* < 0.001). At the 1-year follow-up, data were available for 387 patients in Group 1 and 426 patients in Group 2. Conversely, there were no significant differences between Group 1 and Group 2 in PVR (14.7 mL vs. 14.1 mL, respectively, *p* = 0.23) and Qmax (22.9 mL vs. 26.8 mL, respectively, *p* = 0.16). Additionally, elderly patients exhibited similar IPSS scores compared to their younger counterparts (5.2 vs. 4.8, *p* = 0.06).

Table 3 details postoperative morbidities. Among minor complications, a higher rate of blood transfusion was reported among elderly patients compared to younger ones (4.7% vs. 2.2%, *p* = 0.04). The incidence of other minor and major morbidities was similar between the two groups. Late postoperative morbidities were also comparable. Notably, the incidence of postoperative urinary incontinence was similar between elderly and younger patients at both 1 month and 1 year. However, the rate of Kegel exercise practice was higher in the elderly group (13.8% vs. 9.2%, *p* = 0.02).

The Trifecta Score in the elderly group was achieved in (63.5%) with no statistical difference with the younger counterparts (68.8%, *p* = 0.08). In the multivariate logistic regression analysis, none of the Trifecta Score parameters exhibited a negative association with age (Table 4). Similarly, age was not associated with 1-year postoperative UI, whereas OT (OR 1.05, 95% CI 1.02–1.09, *p* = 0.02) was correlated with higher odds. (Table 5).

## 4. Discussion

As lifespans lengthen and the global elderly population grows, the demand for interventions to treat BPH will inevitably rise, underscoring the need for effective medical and surgical strategies to enhance patient outcomes. Approximately 60% of men with BPH experience a gradual onset of LUTS, often delaying medical consultation until severe and potentially irreversible bladder dysfunction occurs [12,13]. Men over 80 years are particularly vulnerable to renal function impairment due to diminished renal reserve and reduced compensatory mechanisms [14,15]. In addition, elderly patients frequently present with various comorbid conditions, complicating treatment and increasing the risks associated with surgical procedures. This scenario underscores the importance of early detection and comprehensive management strategies [16].

In elderly patients unresponsive to medication, surgical interventions prioritize enhancing functional and voiding outcomes over preserving ejaculatory function, necessitating a carefully tailored approach due to the frailty and anesthesia-related risks in this population. TURP, especially with bipolar technology, remains the gold standard for small to medium-sized prostates, even in elderly patients, while robust scientific evidence for the efficacy of minimally invasive surgical techniques is still lacking [17]. However, while elderly men undergoing TURP or OP face significantly higher postoperative complication rates than younger counterparts, HoLEP provides superior functional outcomes and a lower complication profile, even in older patients with multiple comorbidities [18].

In our results, a higher rate of blood transfusion occurred in the elderly group, who generally exhibited more severe baseline characteristics. This elevated transfusion requirement may be attributed not only to increased frailty and diminished physiological reserves typically associated with advanced age, but also to a greater prevalence of comorbidities necessitating antithrombotic therapies, which collectively exacerbate intraoperative and postoperative bleeding risks. These findings are compatible with the study by Romero-Otero et al., which identified age, prostate size, and antiplatelet/anticoagulant treatments as predictive factors for decreases in hemoglobin and hematocrit [19]. The latter medical therapies occur at a higher rate in Group 1, aligning with the trend shown by the American Geriatrics Society, where the proportion of persons aged ≥80 years on oral anticoagulation increased from 32.4% in 2011 to 43.6% in 2019 [20]. Moreover, age-related changes in vascular integrity and coagulation dynamics may further contribute to the need for transfusions in elderly patients undergoing surgical interventions. Nevertheless, HoLEP is preferable to bipolar TURP as a prostate size-independent treatment option due to its lower rate of blood transfusions and significantly smaller decrease in hemoglobin levels [21].

On the other hand, the aging process impairs innate and adaptive immune responses, increasing older adults’ susceptibility to infections due to comorbidities and age-related declines in immune functions, which can lead to greater morbidity and mortality [22]. However, despite these challenges, the incidence of UTI and sepsis following HoLEP did not increase among the elderly in our study, demonstrating the overall safety of the procedure irrespective of age.

Urinary continence is regulated by a complex physiological system. Although well-established risk factors include physical inactivity, overweight or obesity, and unhealthy dietary habits, normal aging itself is not considered an independent risk factor for incontinence [23]. Consistent with this, a multicenter study conducted by experienced endourologists specializing in endoscopic enucleation of the prostate reported an overall UI rate of 14.8% [24]. In our analysis, postoperative UI rates did not differ significantly between elderly and younger patients, suggesting that age alone does not substantially affect continence outcomes following enucleation. This interpretation is reinforced by our multivariate logistic regression analysis, which demonstrated no independent association between age and UI at 1-year postoperatively. Consequently, advanced age should not be regarded as a contraindication for offering HoLEP to appropriately selected patients. Conversely, OT was identified as a significant predictor of postoperative UI, indicating that prolonged surgical duration may elevate the risk of persistent incontinence. This relationship may reflect increased technical complexity or intraoperative challenges in longer procedures, potentially leading to greater trauma to anatomical structures critical for continence preservation [25]. These findings underscore the importance of surgical efficiency and meticulous technique in minimizing postoperative incontinence, particularly in complex or prolonged procedures.

In terms of functional outcomes, both groups showed improvement in LUTS and uroflowmetry parameters. However, at the 3-month follow-up, Group 1 patients had a higher IPSS and a lower Qmax, indicating a delayed functional recovery, possibly due to physiological detrusor underactivity in the elderly. Notably, the elderly group exhibited a higher preoperative PVR volume than the younger group, as reported even by Savin et al., who found significantly higher rates of chronic urinary retention and catheter use at presentation in octogenarians compared to men under 80 years of age [26]. Nevertheless, 3-month postoperative PVR was similar between two groups.

Nevertheless, evaluating long-term outcomes in elderly patients undergoing HoLEP is essential for determining the procedure’s efficacy in this age group. In a cohort study of 311 patients categorized by age decades, the ≥80-year group exhibited consistently lower Qmax values compared to younger groups, yet showed improvement throughout the follow-up period [27]. Conversely, Piao et al. found that patients aged ≥80 years had a lower Qmax than other age groups at the 3-month follow-up (16.8 vs. 25.1 mL/s, *p* = 0.004), with the results progressively becoming non-significant after 6 months (17.1 vs. 23.6 mL/s, *p* = 0.08) [28]. Similarly, our results indicate that the differences observed in IPSS and Qmax at 3 months were balanced by the 1-year mark, with no significant disparities evident.

The Trifecta Score integrates multiple domains of success into a unified evaluative framework, providing a holistic assessment of procedural efficacy and patient recovery. Its adoption has increasingly become a benchmark in clinical practice and research, as achieving the Trifecta is associated with superior patient satisfaction and long-term therapeutic success. This reporting system has been applied to HoLEP, where it evaluates three critical domains: functional outcomes, surgical efficiency, and safety. However, despite its utility, there is a lack of consensus regarding the precise definitions of its components [29]. To the best of our knowledge, this study represents the first report to specifically report the Trifecta Score of HoLEP for elderly patients and evaluate the impact of age in this surgery. In our analysis, we employed the Trifecta criteria proposed by Autorino et al. [8], which include achieving a Q-max > 15 mL/sec, an IPSS < 8, and the absence of perioperative complications. The Trifecta achievement rate was comparable between elderly patients and their younger counterparts. Additionally, when evaluating individual parameters, no significant differences were observed between the groups. HoLEP demonstrated equivalent efficacy in resolving LUTS and improving Q-max while maintaining low morbidity rates across all age groups. Our findings did not identify age as a risk factor for achieving the Trifecta Score, indicating that HoLEP is an effective option for patients with BPH refractory to medical treatment, regardless of chronological age. However, the three parameters currently included in the Trifecta Score may be insufficient for a comprehensive evaluation of surgical quality. Grosso et al. proposed an alternative set of parameters, emphasizing the importance of UI during postoperative follow-up given its significant impact on patient outcomes [29]. Therefore, while the Trifecta Score remains a valuable tool for assessing the quality of surgical outcomes in HoLEP across all age groups, future evaluations could benefit from incorporating additional information on long-term functional outcomes, such as 1-year postoperative UI and Qmax. This could potentially lead to the development of a Pentafecta Score, as seen in other urological contexts [30]. Nevertheless, further research is required to identify the most appropriate parameters and to establish definitive conclusions.

This study is not without limitations. Firstly, the retrospective nature of this investigation introduces inherent constraints on establishing causal relationships and controlling for potential confounding variables. Secondly, the exclusion of patients with long-term catheter use, catheter-associated UTIs, detrusor underactivity, or overactive bladders limits the generalizability of the findings to these subpopulations. Furthermore, the absence of objective assessments such as urodynamic studies constrains the interpretation of postoperative urinary function, particularly in elderly patients where detrusor underactivity may influence symptom resolution and Qmax outcomes. Thirdly, the results may be limited to the experience of a single center, and potential selection bias may be present in this study. However, it is important to note that the procedures were performed by multiple operators with varying levels of expertise, which may have introduced additional variability. Finally, this study does not provide data on prostate cancer follow-up or the correlation between preoperative and postoperative weight, which could be important factors in understanding the broader implications of the treatment outcomes.

## 5. Conclusions

Based on our study, we conclude HoLEP should be offered to elderly patients for whom the procedure is deemed feasible. Patients should be appropriately counseled to expect a gradual recovery over three months, with functional outcomes by the end of one year likely comparable to those of younger cohorts. Overall, the Trifecta Score appears to be valuable tool for the assessment of HoLEP outcomes in elderly patients. Nevertheless, incorporating assessments of 1-year postoperative Qmax and urinary continence could further enhance the validity of the scoring system. In this context, our findings suggest that age, rather than prolonged operative time, does not significantly increase the risk of UI.

## Figures and Tables

**Table 1:** Patients’ baseline demographics and characteristics. ASA: American Society of Anesthesiologists; IUC: Indwelling Urinary Catheterization; PSA: Prostate-specific antigen; IPSS: International Prostate Symptom Score; Q max: The maximum urinary flow rate; PVR: Post-void Residual.

Variable	Group 1 Age ≥ 75 Years *n* = 490	Group 2 Age < 75 Years *n* = 491	*p* Value
Age, years	78.9 (3.7)	65.1 (6.15)	**<0.001**
Body Mass Index, kg/m^2^	26.0 (2.6)	25.3 (2.7)	**0.008**
ASA Score, *n* (%)			
1	56 (11.4)	148 (30.1)	**<0.001**
2	272 (55.5)	297 (60.5)	0.11
3	162 (33.1)	46 (9.4)	**<0.001**
Charlson Comorbidity Index	4.43 (0.94)	1.75 (0.49)	**<0.001**
Surgery with ongoing anticoagulant/antiplatelet, *n* (%)			**0.004**
Yes	117 (23.8)	81 (16.4)	
No	373 (76.2)	410 (83.6)	
Prostate volume, mL	73.6 (38.9)	74.0 (37.4)	0.92
Preoperative PSA, ng/mL	2.43 (2.17)	2.85 (2.26)	0.39
Preoperative IUC, *n* (%)			0.66
Yes	42 (8.6)	46 (9.3)	
No	448 (91.4)	445 (90.7)	
Preoperative IPSS	20.4 (5.7)	17.9 (2.4)	0.41
Preoperative Q max, mL/s	9.1(2.7)	8.7 (2.9)	0.57
Preoperative PVR, mL	74.7 (55.2)	67.8 (52.1)	**0.01**

**Table 2:** Perioperative characteristics and follow-up analysis of patient symptoms and micturition variables. IPSS: International Prostate Symptom Score; Q max: The maximum urinary flow rate; PVR: Post-void Residual. 3MPO: 3-month postoperative; 1YPO: 1-year postoperative.

Variable	Group 1 Age ≥ 75 Years *n* = 490	Group 2 Age < 75 Years *n* = 491	*p* Value
Operative time, minutes	57.3 (27.5)	54.6 (24.2)	0.26
Enucleation type, *n* (%)			0.26
3 lobes	268 (54.6)	286 (58.3)	
2 lobes	222 (45.4)	205 (41.7)	
Electrocautery after enucleation, *n* (%)	31 (6.3)	109 (5.6)	0.54
Enucleation time, minutes	44.0 (21.4)	43.2 (12.6)	0.71
Adenoma requiring monopolar resection, *n* (%)			0.63
Yes	22 (4.5)	19 (3.8)	
No	468 (95.5)	472 (96.2)	
Length of hospital stay, days	2.03 (0.23)	2.01 (0.13)	0.09
Time to urethral catheter removal, days	2.23 (1.20)	2.07 (0.73)	**0.02**
Histologic findings, *n* (%)			**0.04**
Incidental prostate cancer	27 (5.5)	14 (2.8)	
Benign prostatic tissue	463 (94.5)	477 (97.2)	
No perioperative complications, *n* (%)	424 (86.5)	444 (90.4)	0.06
3MPO IPSS *	7.6 (4.8)	7.0 (4.5)	**0.04**
3MPO IPSS < 8, *n* (%)	346 (70.6)	369 (75.1)	0.11
3MPO Q max *, mL/s	23.6 (5.8)	27.9 (7.2)	**<0.001**
3MPO Q max > 15 mL/s, *n* (%)	448 (91.4)	463 (94.3)	0.08
3MPO PVR *, mL	16.2 (12.3)	14.9 (13.6)	0.05
1YPO IPSS ^∆^	5.2 (3.9)	4.8 (4.2)	0.06
1YPO Q max ^∆^, mL/s	22.9 (4.4)	26.8 (5.1)	0.16
1YPO PVR ^∆^, mL	14.7 (10.5	14.1 (9.6)	0.23
Trifecta Score Achievement, *n* (%)	311 (63.5)	338 (68.8)	0.08

* Data were available for 490 patients in Group 1 and 491 patients in Group 2. ^∆^ Data were available for 387 patients in Group 1 and 426 patients in Group 2.

**Table 3:** Postoperative early and late complications. CD: Clavien–Dindo; ICU: Intensive Care Unit; BPH: Benign Prostate Hyperplasia.

Variable	Group 1 Age ≥ 75 Years *n* = 490	Group 2 Age < 75 Years *n* = 491	*p* Value
**Early complications, *n* (%)**			
Urinary tract infection (CD 2)	17 (3.5)	14 (2.9)	0.58
Acute urinary retention within 24 h (CD 2)	21 (4.3)	16 (3.3)	0.40
Blood transfusion (CD2)	23 (4.7)	11 (2.2)	**0.04**
Postoperative bleeding needing endoscopic hemostasis (CD 3)	6 (1.2)	3 (0.6)	0.31
Sepsis needing ICU (CD 4)	2 (0.4)	2 (0.4)	0.99
**Late complications, *n* (%)**			
Bulbar urethral stricture requiring outpatient dilatation	17 (3.5)	11 (2.3)	0.25
Urethral stricture necessitating urethrotomy under anesthesia	11 (2.2)	13 (2.6)	0.68
Bladder neck sclerosis requiring transurethral Incision	23 (4.7)	14 (2.8)	0.13
Repeated surgery for BPH within 1 year	5 (1.1)	4 (0.8)	0.74
1-Month postoperative urinary incontinence	72 (14.7)	66 (13.4)	0.57
Urge	21 (4.3)	16 (3.3)	0.40
Stress	48 (8.3)	37 (7.5)	0.21
Mixed	10 (2.1)	13 (2.6)	0.53
Kegel exercise needed, *n* (%)	68 (13.8)	45 (9.2)	**0.02**
1-Year postoperative urinary incontinence	36 (7.3)	31 (6.2)	0.52
Urge	2 (0.4)	3 (0.6)	0.66
Stress	31 (6.3)	23 (4.6)	0.26
Mixed	3 (0.6)	5 (1.0)	0.74

**Table 4:** Multivariate logistic regression of Trifecta Score influenced by age. IPSS: International Prostate Symptom Score; Q max: The maximum urinary flow rate.

Parameters	Odds Ratio (95% Confidence Interval)	*p* Values
Perioperative complications	1.23 (0.78–2.96)	0.74
3-month postoperative Qmax (>15 mL/s)	0.76 (0.47–1.52)	0.51
3-month postoperative IPSS (<8)	1.03 (0.58–1.74)	0.83

**Table 5:** Multivariate logistic regression of factors associated with 1-year postoperative urinary incontinence. IPSS: International Prostate Symptom Score; Q max: The maximum urinary flow rate; PVR: Post-void residual.

Parameters	Urinary Incontinence After 1 Year OR (95% CI)	*p* Values
Age	0.98 (0.95–1.04)	0.55
Prostate Volume	0.95 (0.92–1.08)	0.36
Preoperative Qmax	0.91 (0.81–0.94)	0.38
Preoperative IPSS	1.06 (1.01–1.13)	0.92
Preoperative PVR	0.88 (0.81–0.95)	0.07
Operative time	1.05 (1.02–1.09)	**0.02**
Length of Stay	0.91 (0.53–1.86)	0.65

## Data Availability

The dataset used in this study is available upon request from the corresponding author.

## Abbreviations

BPH	Benign Prostatic Hyperplasia
LUTS	Lower Urinary Tract Symptoms
EAU	European Association of Urology
TURP	Transurethral Resection of the Prostate
OP	Open Prostatectomy
OT	Operative Time
HoLEP	Holmium Laser Enucleation of the Prostate
ASA	American Society of Anesthesiologists
Qmax	Maximum Urinary Flow Rate
IPSS	International Prostate Symptom Score
ET	Enucleation Time
LOS	Length of Hospital Stay
CD	Clavien–Dindo
PSA	Prostate-Specific Antigen
CCI	Charlson Comorbidity Index
UI	Urinary Incontinence
OR	Odds Ratio
CI	Confidence Interval
UTI	Urinary Tract Infection

## References

[1] D’Agate S., Wilson T., Adalig B., Manyak M., Palacios-Moreno J.M., Chavan C., Oelke M., Roehrborn C., Della Pasqua O. (2020). Impact of disease progression on individual IPSS trajectories and consequences of immediate versus delayed start of treatment in patients with moderate or severe LUTS associated with BPH. World J. Urol..

[2] Bartoletti R., Cai T., Tinacci G., Longo I., Ricci A., Massaro M.P., Tosoratti N., Zini E., Pinzi N. (2008). Transperineal microwave thermoablation in patients with obstructive benign prostatic hyperplasia: A phase I clinical study with a new mini-choked microwave applicator. J. Endourol..

[3] Madersbacher S., Sampson N., Culig Z. (2019). Pathophysiology of Benign Prostatic Hyperplasia and Benign Prostatic Enlargement: A Mini-Review. Gerontology.

[4] Rhodes T., Girman C.J., Jacobsen S.J., Roberts R.O., Guess H.A., Lieber M.M. (1999). Longitudinal prostate growth rates during 5 years in randomly selected community men 40–79 years old. J. Urol..

[5] Cornu J.N., Gacci M., Hashim H., Herrmann T.R.W., Malde S., Netsch C., De Nunzio C., Rieken M., Sakalis V., Tutolo M. (2024). EAU Guidelines on Non-Neurogenic Male Lower Urinary Tract Symptoms (LUTS).

[6] Scarcella S., Castellani D., Gauhar V., Teoh J.Y.-C., Giulioni C., Piazza P., Bravi C.A., De Groote R., De Naeyer G., Puliatti S. (2021). Robotic-assisted versus open simple prostatectomy: Results from a systematic review and meta-analysis of comparative studies. Investig. Clin. Urol..

[7] Tallè M., Giulioni C., Papaveri A., Mengoni F., Orciani R., Pandolfo S.D., Imbimbo C., Crocetto F., Castellani D., Herrmann T. (2025). Influence of preoperative indwelling urinary catheter on outcomes of high-power holmium laser enucleation for very large prostate (≥200 mL). World J. Urol..

[8] Yilmaz M., Esser J., Suarez-Ibarrola R., Gratzke C., Miernik A. (2022). Safety and Efficacy of Laser Enucleation of the Prostate in Elderly Patients—A Narrative Review. Clin. Interv. Aging.

[9] Autorino R., Zargar H., Mariano M.B., Sanchez-Salas R., Sotelo R.J., Chlosta P.L., Castillo O., Matei D.V., Celia A., Koc G. (2015). Perioperative Outcomes of Robotic and Laparoscopic Simple Prostatectomy: A European-American Multi-institutional Analysis. Eur. Urol..

[10] World Health Organization (2015). Significant loss of functional ability, and care dependence. World Rep. Aging Heal..

[11] Gilling P.J., Kennett K., Das A.K., Thompson D., Fraundorfer M.R. (1998). Holmium laser enucleation of the prostate (HoLEP) combined with transurethral tissue morcellation: An update on the early clinical experience. J. Endourol..

[12] Mantica G., Ambrosini F., Drocchi G., Zubko Z., Monaco L.L., Cafarelli A., Calarco A., Colombo R., De Cobelli O., De Marco F. (2024). Non-surgical management of BPH: An updated review of current literature and state of the art on natural compounds and medical therapy. Arch. Ital. Urol. Androl..

[13] Palumbo S., Lucarelli G., Lasorsa F., Damiano R., Autorino R., Aveta A., Spena G., Perdonà S., Russo P., Giulioni C. Urobiome and Inflammation: A Systematic Review on Microbial Imbalances and Diagnostic Tools for Urinary Disorders. Urology.

[14] O’Sullivan E.D., Hughes J., Ferenbach D.A. (2017). Renal Aging: Causes and Consequences. J. Am. Soc. Nephrol..

[15] Giulioni C., Palantrani V., De Stefano V., Cicconofri A., Antezza A., Beltrami M., Milanese G., Ranghino A., Gauhar V., Castellani D. (2023). Current Evidence on Surgical Management for Benign Prostatic Hyperplasia in Renal Transplant Recipients: A Systematic Review. J. Endourol..

[16] Wang Q., Zhang B., Li B., Yang S., Wang Z., Han C., Wu J., Tian R. (2023). Correlation Between Benign Prostatic Hyperplasia/Lower Urinary Tract Symptoms and Renal Function in Elderly Men Aged 80 Years and Older. Clin. Interv. Aging.

[17] Brandt T.W., Luizzi J.M., Caras R.J. (2024). Evaluation of Current Surgical BPH Interventions for Young and Elderly Men. Curr. Urol. Rep..

[18] Burtt G., Springate C., Martin A., Woodward E., Zantek P., Al Jaafari F., Muir G., Misrai V. (2022). The Efficacy and Safety of Laser and Electrosurgical Transurethral Procedures for the Treatment of BPO in High-Risk Patients: A Systematic Review. Res. Rep. Urol..

[19] Romero-Otero J., García-González L., García-Gómez B., Justo-Quintas J., García-Rojo E., González-Padilla D.A., Sopeña-Sutil R., Duarte-Ojeda J.M., Rodríguez-Antolín A. (2019). Factors Influencing Intraoperative Blood Loss in Patients Undergoing Holmium Laser Enucleation of the Prostate (HoLEP) for Benign Prostatic Hyperplasia: A Large Multicenter Analysis. Urology.

[20] Harrison S.L., Buckley B.J.R., Ritchie L.A., Proietti R., Underhill P., Lane D.A., Lip G.Y.H. (2022). Oral anticoagulants and outcomes in adults ≥80 years with atrial fibrillation: A global federated health network analysis. J. Am. Geriatr. Soc..

[21] Daryanto B., Suryanullah W.S., Putra P.Y.P. (2025). Holmium laser enucleation of the prostate versus transurethral resection of the prostate in treatment of benign prostatic hyperplasia: A meta-analysis of 13 randomized control trials. Curr. Urol..

[22] Agarwal S., Busse P.J. (2010). Innate and adaptive immunosenescence. Ann. Allergy Asthma Immunol..

[23] Olagundoye O., Ross S., Gibson W., Wagg A. (2024). Defining and prioritizing modifiable risk factors towards the co-creation of a urinary incontinence self-management intervention for older men: A sequential multimethod study protocol. PLoS ONE.

[24] Gauhar V., Sancha F.G., Enikeev D., Sofer M., Fong K.Y., Socarrás M.R., Elterman D., Chiruvella M., Bendigeri M.T., Tursunkulov A.N. (2023). Results from a global multicenter registry of 6193 patients to refine endoscopic anatomical enucleation of the prostate (REAP) by evaluating trends and outcomes and nuances of prostate enucleation in a real-world setting. World J. Urol..

[25] Fan X., Zhang J., Zhu H., Huang F., Shadike A., Jiang C. (2024). Predictive factors of stress urinary incontinence after Holmium Laser Enucleation of the Prostate: A magnetic resonance imaging-based retrospective study. Transl. Androl. Urol..

[26] Savin Z., Veredgorn Y., Taha T., Alsaraia N., Lifshitz K., Nevo A., Yossepowitch O., Sofer M. (2023). En bloc holmium laser enucleation of prostate in octogenarians and nonagenarians: Clinical characteristics and outcome. Lasers Med. Sci..

[27] Mmeje C.O., Nunez-Nateras R., Warner J.N., Humphreys M.R. (2013). Age-stratified outcomes of holmium laser enucleation of the prostate. BJU Int..

[28] Piao S., Choo M.S., Kim M., Jeon H.J., Oh S.J. (2016). Holmium Laser Enucleation of the Prostate is Safe for Patients Above 80 Years: A Prospective Study. Int. Neurourol. J..

[29] Grosso A.A., Di Maida F., Nardoni S., Salvi M., Giudici S., Lambertini L., Cadenar A., Tellini R., Cocci A., Mari A. (2023). Patterns and Predictors of Optimal Surgical and Functional Outcomes after Holmium Laser Enucleation of the Prostate (HoLEP): Introducing the Concept of “Trifecta”. World J. Mens. Health..

[30] Asimakopoulos A.D., Miano R., Di Lorenzo N., Spera E., Vespasiani G., Mugnier C. (2013). Laparoscopic versus robot-assisted bilateral nerve-sparing radical prostatectomy: Comparison of pentafecta rates for a single surgeon. Surg. Endosc..

